# *Lactobacillus iners* and vaginal microbiota diversity as risk factors of uterine cervix dysplasia: a prospective study

**DOI:** 10.3389/frph.2026.1797643

**Published:** 2026-03-27

**Authors:** Tomas Rokos, Veronika Holubekova, Lucia Mackova, Erik Kozubik, Marian Grendar, Lucia Kotulova, Andrea Hornakova, Zuzana Kolkova, Terezia Pribulova, Elena Novakova, Kamil Biringer, Erik Kudela

**Affiliations:** 1 Department of Gynecology and Obstetrics, Jessenius Faculty of Medicine in Martin Comenius University in Bratislava (JFMCU) and University Hospital Martin (UHM), Martin, Slovakia; 2 Laboratory of Genomics and Prenatal Diagnostics, Biomedical Center Martin (BIOMED), Jessenius Faculty of Medicine in Martin Comenius University in Bratislava (JFMCU), Martin, Slovakia; 3 Laboratory of Bioinformatics and Biostatistics, Biomedical Center Martin (BIOMED), Jessenius Faculty of Medicine in Martin Comenius University in Bratislava (JFMCU), Martin, Slovakia; 4 Department of Microbiology and Immunology, Jessenius Faculty of Medicine in Martin Comenius University in Bratislava (JFMCU), Martin, Slovakia

**Keywords:** cervical dysplasia, HPV, lactobacilli, lactobacillus iners, vaginal microbiota

## Abstract

**Purpose:**

The aim of this study was to observe the vaginal microbiota composition in patients with cervical dysplasia.

**Methods:**

In this prospective study, 91 samples for vaginal microbial diversity examination were taken from the cervix and posterior vaginal fornix. Eighteen bacterial species, including *Lactobacillus* species, were identified by real-time PCR. Relative bacterial quantities (RQs) were calculated, and log2-transformed RQs were visualised with boxplots. The relationships between cytological abnormalities and bacterial species were calculated by nonparametric one-way ANOVA (Kruskal–Wallis test). For determination of bacterial diversity, the Shannon index was used.

**Main finding:**

We demonstrated an increase in the abundance of vaginal microbes dominated by *L. iners* with increasing severity of cytologically and histologically confirmed cervical lesions, while HPV infection was present in 73.1% of community state type 3 samples. The presence of *G. vaginalis* and *U. parvum* in combination with *L. iners* was statistically significant. Our study also revealed considerably higher bacterial diversity in the community state type IV category. An increasing trend of bacterial diversity with increasing cytological severity of cervical lesions was also observed, although the difference was nonsignificant.

**Conclusion:**

This study suggests that *L. iners* has adverse effects on the development of cervical dysplasia, while *L. gasseri* and *L. crispatus* may play protective roles. We also pointed out the association of *L. iners* with anaerobic bacteria, and we suggest the potential role of bacterial diversity in cervical carcinogenesis.

## 1 Introduction

The human body is colonised by a vast array of microorganisms that collectively form the human microbiome. It comprises approximately 500–1,000 bacterial species (spp.)  (1), with a total gene content that exceeds that of the human genome  (2). The vaginal microbiome, which is predominantly composed of *Lactobacillus* spp., plays a crucial role in preventing urinary tract infections  (3), urogenital disorders, and sexually transmitted infections  (4).

The vaginal microbiota of sexually active women can be classified into five distinct community state types (CSTs). Four of these (CSTs I, II, III, and V) are dominated by *Lactobacillus* species—*Lactobacillus crispatus* (CST I), *L. gasseri* (CST II), *L. iners* (CST III), and *L. jensenii* (CST V). In contrast, CST IV is characterised by a depletion of lactobacilli and a dominance of strict anaerobes (5). *Lactobacillus* species are considered protective against colonisation by vaginal pathogens because of their production of organic acids, hydrogen peroxide (H_2_O_2_), and antimicrobial peptides such as bacteriocins (6). Conversely, the overgrowth of anaerobic bacteria—a condition referred to as *bacterial vaginosis (BV)*—is associated with reduced lactate levels and increased concentrations of short-chain fatty acids (SCFAs) and their anions. Unlike those in the intestinal microbiota, elevated SCFA levels in the vaginal environment are indicative of dysbiosis (7).

A shift from *Lactobacillus*-dominant vaginal microbiota to pathogen-dominant communities—indicative of vaginal dysbiosis—leads to disruptions in immune function and epithelial homeostasis. This transition is associated with increased production of proinflammatory cytokines and chemokines, activation of immune pathways, and reduced viscosity of cervicovaginal secretions due to mucin-degrading enzyme activity. These changes compromise mucosal barrier integrity, thereby increasing susceptibility to sexually transmitted infections, including high-risk human papillomavirus (hrHPV) infections (8).

Interestingly, not all *Lactobacillus* species play a clearly protective role. *L. iners*, first described in 1,999 (9), is among the most prevalent vaginal species (10). However, its presence is not consistently associated with a healthy microbiota. Srinivasan et al. (11) reported that women with high levels of *L. crispatus* rarely have BV, whereas those with high levels of *L. iners* could be either BV-negative or BV-positive. These findings suggest that *L. iners* may not provide sufficient protection against BV (11). Nonetheless, *L. iners* has been demonstrated to have an inhibitory effect on *Gardnerella vaginalis* growth, indicating that it may still play a nuanced role in microbial interactions (12).

Various aspects of vaginal health have been associated with *L. iners* dominance in the vaginal microbiota, as well as with the detrimental effects of vaginal dysbiosis. In our study, we focused on the relationship between variations in the vaginal microbiota composition and the potential development of cervical dysplasia—a topic of continued relevance due to its risk of progression to cervical cancer.

## 2 Materials and methods

### 2.1 Sample collection

Samples were collected from patients with cervical cytological abnormalities who underwent expert colposcopic examination and from healthy controls at the Ambulance of Expert Colposcopy at the Department of Gynaecology and Obstetrics, Jessenius Faculty of Medicine, Comenius University in Bratislava and University Hospital Martin. The examined group included 20 patients with atypical squamous cells with undetermined significance (ASCUS), 25 patients with low-grade squamous intraepithelial lesions (LSILs), and 26 patients with high-grade squamous intraepithelial lesions (HSILs); 20 samples from women who were negative for intraepithelial lesions or malignancies (NILM) constituted the control group. During the examination, a cervical swab was taken from the exocervix and posterior vaginal fornix. The samples were transported to the Laboratory of Genomics and Prenatal Diagnostics, Biomedical Centre Martin, Jessenius Faculty of Medicine, Comenius University, in Bratislava and stored at −80°C in viral/bacterial transport medium (Jiangsu Mole Bioscience, Taizhou, China) until further processing. The study was performed in accordance with the Declaration of Helsinki and was approved by the Ethics Committee of the Jessenius Faculty of Medicine in Martin, Comenius University in Bratislava no. EK 85/2020 with obtained informed consent from all individual participants included in the study.

### 2.2 Nucleic acid extraction

The collected samples were thawed and mixed before processing. The samples were subsequently centrifuged at 3,000 revolutions per minute (rpm) for 10 min at 4 °C. The supernatant was removed, and the cells were washed twice with phosphate-buffered saline (pH 7.0). Subsequently, 100 μl of the cell suspension was used for DNA extraction with the MasterPure Complete DNA and RNA Purification Kit (Biosearch Technologies, Hoddesdon, UK) according to the manufacturer's instructions. The extracted DNA was incubated with RNase A for 30 min at 37 °C. The deoxyribonucleic acid (DNA) was diluted in 35 μl of TE (Tris + EDTA, pH 8) buffer. The quality and quantity of the DNA were measured with a Qubit dsDNA HS assay kit (Invitrogen, Thermo Fisher Scientific, Carlsbad, CA, USA) on a Qubit 4.0 instrument (Invitrogen).

## 3 Detection of selected microbial strains

We tested 120 ng of DNA by real-time polymerase chain reaction (qPCR) to identify eighteen microbial strains according to the manufacturer's instructions (Custom Microbial DNA qPCR Array, Qiagen, Hilden, Germany). The PCR plate contained microbial DNA arrays of 18 bacterial species (*L. crispatus*, *L. gasseri*, *L. jensenii*, *L. iners*, *Aerococcus christensenii*, *Atopobium vaginae*, *Fusobacterium nucleatum*, *G. vaginalis*, *Leptotrichia amnionii*, *Mobiluncus* spp., *Mycoplasma hominis*, *Parvimonas micra*, *Prevotella bivia*, *Prevotella disiens*, *Sneathia sanguinegens*, *Streptococcus agalactiae*, *Streptococcus mitis*, and *Ureaplasma parvum*), bacterial 16S rRNA (Pan B1 and Pan B3) and fungal ribosomal rRNA genes (Pan Asp/Can), and a positive PCR control (PPC). One plate allowed the examination of four patients. In total, 25 microL (μl) of a solution consisting of 12.5 μl of microbial qPCR master mix, primer/probe depending on the position on the plate, and 11.5 μl of microbial DNA-free water (Qiagen) together with 5 ng of DNA was applied to the wells of a 96-well plate.

The microbial strains were detected with a QuantStudio 5 (Applied Biosystems) with thermal cycling with initial denaturation at 95 °C for 10 min, an additional 40 cycles of denaturation at 95 °C for 15 s and annealing and elongation at 60 °C for 2 min. All the samples were analysed with the instrument software according to the manufacturer's recommendations.

HPV infection was identified according to a well-established method published in our previous study (13).

## 4 Statistical analysis

For data analysis, R (version 4.4.2) (14) was used.

For this purpose, values were summarised using counts and percentages for categorical variables; for continuous variables, medians with lower and upper quartiles were reported. The threshold for a positive Ct result was set to 37 cycles according to the manufacturer's recommendations.

To inspect the co-occurrence (prevalence) of *L. iners* with other variables across all CST types, Fisher's exact test was used. A subset of CST 4 was subsequently used to examine the co-occurrence of *G. vaginalis* with other bacterial taxa using Fisher's exact test. A one-sample binomial test was applied to examine whether the prevalence of each bacterial taxon differed significantly from the threshold value of 0.5. Owing to multiple testing in this section, Benjamini‒Hochberg correction for *p* value adjustment was applied.

The prevalence of HPV infection between CST 3 and other types (1, 2, and 5) and between CST 4 and other types (1, 2, and 5) was examined. For this purpose, owing to the sufficient number of observations (*n* > 5 in each group), Pearson's chi-square test was used. The resulting *p*-values were also adjusted using the Benjamini–Hochberg correction method.

The Shannon diversity index was computed from relative abundances (abundances were calculated as 2^(-*Δ*Ct), where the average Ct of 16S RNA genes was subtracted from the Ct of the selected bacterium in a given sample). The results were visualised via boxplots at the lesion and CST types. First, the Kruskal‒Wallis test was applied to assess differences among all levels; subsequently, pairwise comparisons between levels were performed using the Wilcoxon rank-sum test. *P*-values for the Kruskal–Wallis tests and for the pairwise comparisons were adjusted using the Benjamini–Hochberg correction method within their respective families of tests. For the CST type, CST 5 was excluded from pairwise comparisons because of the low number of observations. Finally, heatmaps of relative abundances were generated for each sample at each level of the CST type.

## 5 Results

### 5.1 Analysis of clinicopathological data

Among the 91 samples, the average age was 36.2 ± 9.61 years. The presence of HPV infection was detected in 55% (50/91) of the cervical cytological samples and increased with increasing severity of squamous intraepithelial lesions (*p* < 0.05) (Table 1).

**Table 1:** Classification of samples in the CST classes with other monitored parameters.

Characteristic	n	CST 1	CST 2	CST 3	CST 4	CST 5
*n*	91	19	11	26	30	5
Age						
*^1^* median (Q1, Q3)	91	35 (29, 43)	33 (31, 40)	37 (29, 44)	36 (29, 45)	33 (30, 34)
*^1^* mean (SD)	91	37.11 ± 10.75	36.45 ± 8.74	36.08 ± 9.78	38.48 ± 14.37	33.40 ± 9.42
HPV						
Absent, *n* (%)	42	10 (53%)	7 (64%)	7 (27%)	15 (50%)	3 (60%)
Present, *n* (%)	49	9 (47%)	4 (36%)	19 (73%)	15 (50%)	2 (40%)
Lesion						
NILM, *n* (%)	20	5 (26%)	5 (45%)	2 (7.6%)	7 (23%)	1 (20%)
ASCUS, *n* (%)	20	4 (21%)	1 (9.1%)	6 (23%)	7 (23%)	2 (40%)
LSIL, *n* (%)	25	7 (37%)	2 (18.2%)	8 (31%)	8 (27%)	0 (0%)
HSIL, *n* (%)	26	3 (16%)	3 (27.3%)	10 (38.4%)	8 (27%)	2 (40%)
Biopsy						
Negative, *n* (%)	14	8 (42%)	0 (0%)	2 (8%)	3 (10%)	1 (20%)
CIN1, *n* (%)	10	1 (5%)	0 (0%)	4 (15%)	4 (13%)	1 (20%)
CIN2, *n* (%)	22	2 (11%)	2 (18%)	10 (38%)	8 (27%)	0 (0%)
CIN3/CIS, *n* (%)	13	0 (0%)	2 (18%)	5 (19%)	4 (13%)	2 (40%)
Not investigated, *n* (%)	32	8 (42%)	7 (64%)	5 (19%)	11 (37%)	1 (20%)

n, number of samples; CST, community state type; Q, quartile; SD, standard deviation; HPV, human papillomavirus; %, percentage; NILM, negative for intraepithelial lesion or malignancy; ASCUS, atypical squamous cells of undetermined significance; LSIL, low-grade squamous intraepithelial lesion; HSIL, high-grade squamous intraepithelial lesion; CIN, cervical intraepithelial neoplasia.

In this study, we examined the selected microbial strains frequently present in the cervical cytological smears of women in the Zilina region in Slovakia. On the basis of the results of the PCR analysis, the cervical swab samples were classified into CSTs according to the lowest delta Ct number, which was calculated as the Ct of average 16S rRNA genes subtracted from the Ct of the selected bacterium.

In our cohort, 19 samples were classified into the CST 1 category with a dominance of *L. crispatus*, 11 samples were classified into the CST 2 category with a dominance of *L. gasseri*, 26 samples were classified into the CST 3 category with a dominance of *L. iners*, 5 samples were classified into the CST 5 category with a dominance of *L. jensenii*, and 30 samples were classified into the CST 4 category with dominance of bacteria other than *Lactobacillus* spp. There were no significant differences in age among the patients in the categories of CST 1–5 (*p* > 0.9). The division of samples into CST classes with other monitored parameters is shown in Table 1. The proportions of bacterial strains across CST classes are listed in Table 2.

**Table 2:** Representation of bacterial strains among community state type (CST) classes with the number of samples of batcterial strains presentation and percentage representations.

Characteristic	CST 1 *n* (%)	CST 2 *n* (%)	CST 3 *n* (%)	CST 4 *n* (%)	CST 5 *n* (%)
*n*	19	11	26	30	5
Lactobacillus crispatus	19 (100%)	3 (27%)	12 (46%)	9 (30%)	1 (20%)
Lactobacillus gasseri	7 (37%)	11 (100%)	8 (31%)	4 (13%)	2 (40%)
Lactobacillus jensenii	9 (47%)	1 (9%)	14 (54%)	5 (17%)	5 (100%)
Lactobacillus iners	9 (47%)	3 (27%)	26 (100%)	17 (57%)	3 (60%)
Aerococcus christensenii	1 (5%)	0 (0%)	5 (19%)	11 (37%)	1 (20%)
Atopobium vaginae	2 (11%)	3 (27%)	6 (23%)	20 (67%)	2 (40%)
Fusobacterium nucleatum	1 (5%)	1 (9%)	1 (3.8%)	10 (33%)	0 (0%)
Gardnerella vaginalis	8 (42%)	3 (27%)	18 (69%)	25 (83%)	2 (40%)
Leptotrichia amnionii	0 (0%)	0 (0%)	3 (12%)	12 (40%)	0 (0%)
Mobiluncus spp.	2 (11%)	0 (0%)	2 (8%)	10 (33%)	0 (0%)
Mycoplasma hominis	0 (0%)	1 (9%)	1 (4%)	6 (20%)	0 (0%)
Parvimonas micra	1 (5%)	0 (0%)	2 (8%)	15 (50%)	1 (20%)
Prevotella bivia	6 (32%)	3 (27%)	8 (31%)	16 (53%)	3 (60%)
Prevotella disiens	3 (16%)	0 (0%)	2 (8%)	13 (43%)	2 (40%)
Sneathia sanguinegens	0 (0%)	0 (0%)	2 (8%)	13 (43%)	0 (0%)
Streptococcus agalactiae	0 (0%)	5 (45%)	3 (12%)	7 (23%)	3 (60%)
Streptococcus mitis	4 (21%)	1 (9%)	4 (15%)	4 (13%)	0 (0%)
Ureaplasma parvum	7 (37%)	2 (18%)	15 (58%)	8 (27%)	3 (60%)
Pan Aspergillus/Candida	3 (16%)	2 (18%)	5 (19%)	8 (27%)	1 (20%)

n, number of samples; CST, community state type, %, percentage.

### 5.2 Analysis of selected bacterial strains in protective cervical flora (CST 1, CST 2 and CST 5)

Overall, 19 samples that were dominated by *L. crispatus* were categorised as CST 1. In terms of cytological classification, for the CST 1, the results were as follows: normal cytological samples (NILM), 26.3%; ASCUS samples, 21.1%; LSIL samples, 36.8%; and HSIL samples, 15.8%. HPV infection was present in 47.4% of the samples. In patients who underwent biopsy (57.9%), most biopsies were negative (42.1%) for any morphological changes, or CIN1 or CIN2 histological findings (15.8%) were present.

*L. gasseri* was dominant in 11 cytological samples, which were categorised as CST 2. In terms of cytological classification, 45.4% of the samples were NILM, 9.1% were ASCUS samples, 18.2% were LSIL samples, and 27.3% were HSIL samples. HPV infection was present in 36.4% of the samples. In patients who underwent biopsy (36.4%), moderate or severe histological outcomes were equally common in both groups in 18.2% of the samples.

The CST 5 category, dominated by *L. jensenii*, was the smallest group, with 5 samples. Therefore, this group was excluded from further analysis because the sample size was too small.

### 5.3 Analysis of selected bacterial strains in the CST 3 category

A total of 26 cervical swab samples were assigned to the CST 3 category on the basis of having the lowest deltaCt of *L. iners*. The median age of patients in the CST 3 category was 36 years, with lower and upper quartiles of 29 and 44 years, respectively. The mean age of patients was 36.08 years, with a standard deviation of 9.78 years.

HPV infection was present in 73.1% of the CST 3 samples; this result was significantly different (*p* = 0.038) from those of the CST 1, 2 and 5 categories, the bacterial flora of which are dominated by protective *Lactobacillus* spp. In terms of cytological classification, 7.6% of the samples were NILM, 23.1% were ASCUS samples, 30.7% were LSIL samples, and 38.4% were HSIL samples; these results indicate that the CST 3 category is associated with increased lesion severity.

Biopsy was investigated in 80.8% of the CST 3 cervical samples, where 7.7% of the samples were negative for any morphological changes. CIN1 histological findings were present in 15.2% of the samples, CIN2 was present in 38.4% of the samples, and severe histological changes (CIN3/CIS) were present in 19.2% of the CST 3 samples.

We also tested the presence of other bacteria in the CST 3 group. Other bacteria, such as *G. vaginalis* (69%), *U. parvum* (58%), *L. jensenii* (54%), *L. crispatus* (46%), *L. gasseri* (31%) and *P. bivia* (31%), were present in more than 30% of the CST3 samples. Among these bacteria, the presence of *L. gasseri*, *G. vaginalis* and *P. bivia* was marginally significant in the raw (*p* = 0.076) and adjusted (*p* = 0.091) *p* values in more than 30% of the tested samples.

### 5.4 Analysis of selected bacterial strains in the CST4 category

A total of 30 cervical samples were classified into the CST 4 category on the basis of having the lowest deltaCt of bacteria other than *Lactobacillus* spp. The median age of patients in the CST 4 category was 36 years, with lower and upper quartiles of 29 and 45 years, respectively. The mean age of patients was 38.48 years, with a standard deviation of 14.37 years.

Overall, fifty percent of CST 4 samples were positive for HPV infection, which was not significantly different (*p* = 0.6) from the positivity rates of the CST 1, 2 and 5 categories, which had protective cervical flora. NILM or ASCUS were present in 23.3% of the CST4 samples in both groups (HPV-infected and non-HPV-infected). LSIL or HISL cytological findings were detected in 26.7% of the CST4 samples in both groups.

Biopsy was investigated in 63.4% of the CST 4 cervical samples, and 10.0% of the samples were negative for any morphological changes, 13.4% of the samples were classified as CIN1, CIN2 was identified in 26.7% of the samples, and severe histological changes (CIN3/CIS) were identified in 13.3% of the samples.

The most common bacterium in the CST 4 samples was *G. vaginalis*, which was dominant in 56.7% (17/30) of samples and present in 83.3% (25/30) of samples. Other bacterial strains were also dominant in the remaining CST4 samples: *L. amnionii* (13.3%, 4 of 30 samples), *S. agalactiae* (10.0%, 3 of 30 samples), *A. vaginae* (6.7%, 2 of 30 samples), *Candida* spp. (6.7%, 2 of 30 samples), *P. bivia* (3.3%, one of 30 samples) and *S. mitis* (3.3%, one of 30 samples). The bacterial strains present in at least 30% of the CST4 samples included A*. vaginae* (67%), *L. iners* (57%), *P. bivia* (53%), *P. micra* (50%), *P. disiens* (43%), *S. sanguinegens* (43%), *L. amnionii* (40%), *A. christensenii* (37%), *F. nucleatum* (33%), *Mobiluncus* spp. (33%) and *L. crispatus* (30%).

We also investigated how other bacteria co-occur with *G. vaginalis*. Its presence was linked to an approximately 11.5-fold greater chance of detecting *A. vaginae* (raw *p* value = 0.0058; adjusted *p* value = 0.1043). On the other hand, samples with *G. vaginalis* presented a 25-fold lower chance (OR = 0.041) of containing *S. agalactiae* (raw *p* value = 0.0312; adjusted *p* value = 0.2812). These findings suggest possible positive and negative associations, which should be confirmed in a larger patient cohort. The bacterial composition of CST4 is listed in Table 2.

### 5.5 Diversity of bacterial strains across the CST categories

In accordance with our previous study (13), we selected bacterial strains that are frequently present in the cervical swabs of Slovak women in the Zilina region. The strain was evaluated as positive when the Cycle threshold (Ct) was lower than 37 according to the manufacturer's recommendations. A representation of the bacterial strains in the CST classes is shown in Table 2.

To reach our goal, we performed a prevalence analysis of *L. iners* with other bacteria across all CST types to investigate the relationships among the bacterial species.

Overall, we found a more than four-fold (OR = 4.33) greater coexistence with *G. vaginalis* (*p* = 0.0016), which was also a significant difference after *p* value adjustment (*p* = 0.03). We also found more than a three-fold (OR = 3.42) greater (*p* = 0.014) coexistence of *U. parvum* and three-fold (OR = 0.333) lower (*p* = 0.022) coexistence of *L. gasseri* with *L. iners*. The coexistence of both bacteria did not significantly differ after *p* value adjustment (*p* = 0.123 and *p* = 0.131, respectively).

**Figure 1:**
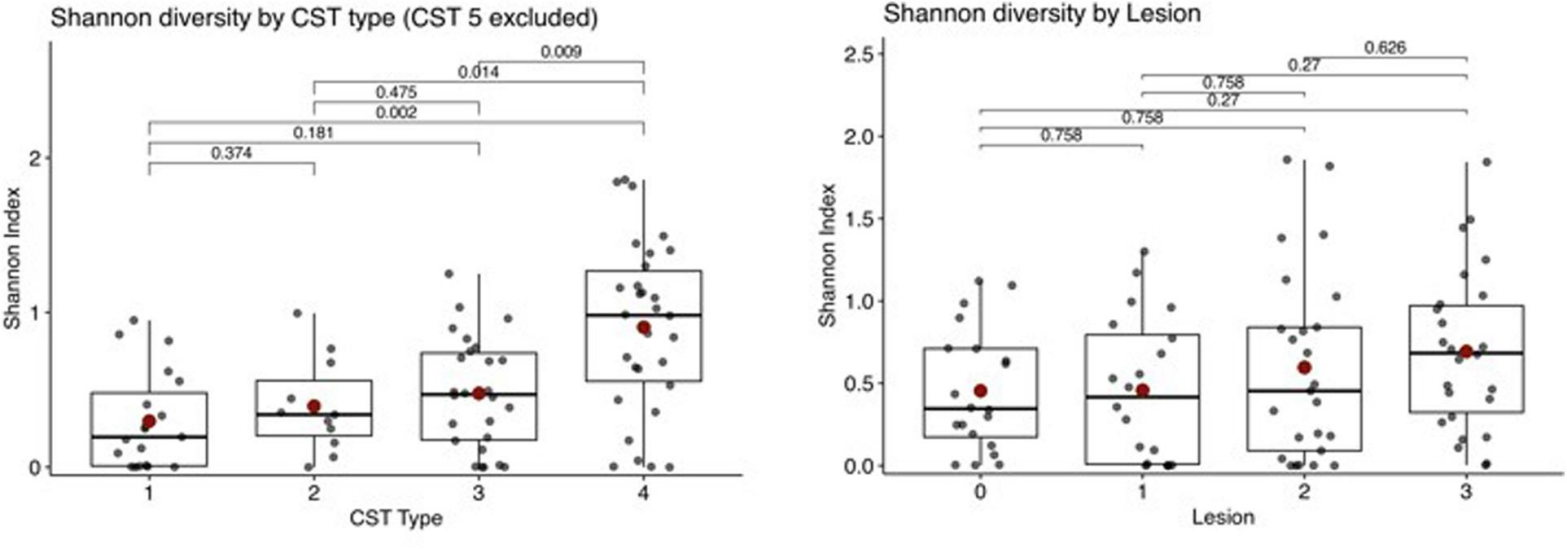
The shannon ndiversity index was used to calculate the relative bacterial abundance across CST types and lesions (0 means NILM, 1 means ASCUS, 2 means LSIL, and 3 means HSIL) the figures report Benjamini–hochberg-adjusted *p*-values for pairwise comparisons.

The bacterial diversity of all the CST classes in the cytological samples was also estimated using the Shannon diversity index. Shannon diversity differed significantly across CST classes (Kruskal–Wallis test, *p* = 0.0012; Table 3), with the highest observed values in CST4. With respect to cytological abnormalities, we observed an increasing trend in the severity of lesions, although the increase was not significant (Table 3). Boxplots with calculated *p* values between the CST classes and lesions are shown in Figure 1. The relative abundances of selected bacterial species across samples in the CST classes are visualised in Supplementary Figures S2–S6.

**Table 3:** Shannon diversity index.

Category	*n*	Shannon diversity index (median, Q1, Q3)	Adjusted *P* value
CST class			
CST1	19	0.19 (0.01, 0.56)	0.0012
CST2	11	0.34 (0.16, 0.68)	
CST3	26	0.47 (0.17, 0.75)	
CST4	30	0.98 (0.53, 1.30)	
CST5	5	0.11 (0.09, 0.71)	excluded
Cytology			
NILM	20	0.35 (0.16, 0.71)	0.2776
ASCUS	20	0.42 (0.01, 0.82)	
LSIL	25	0.45 (0.09, 0.84)	
HSIL	26	0.68 (0.30, 0.98)	

n, number of samples; CST, community state type; Q, quartile; SD, standard deviation; HPV, human papillomavirus; %, percentage; NILM, negative for intraepithelial lesion or malignancy; ASCUS, atypical squamous cells of undetermined significance; LSIL, low-grade squamous intraepithelial lesion; HSIL, high-grade squamous intraepithelial lesion; CIN, cervical intraepithelial neoplasia.

## 6 Discussion

*Lactobacillus* spp. present in the vaginal flora are regarded as bacteria with a protective effect on epithelial integrity. As mentioned in the introduction, vaginal/cervical samples can be divided into categories according to microbial composition, and these categories potentially have different risks of squamous intraepithelial lesion development. The aims of our study were to examine the microbial composition and compare CST and cytological classification. An additional aim was to explore the relationship between *L. iners* and other selected bacteria.

Our study involved the use of cervical swabs/samples taken from Slovak women with abnormal results and from those with normal results of cervical cytology. After investigating the cervical microbiota composition, we categorised the samples into main classes: CST 1, CST 2, CST 3, CST 4 and CST 5. The most common class was CST 4 (30/91, 33.0%), followed by CST3 (26/91, 28.6%), CST1 (19/91, 20.9%), CST2 (11/91, 12.1%) and CST5 (5/91, 5.5%). The same order of abundance of CST categories was found in a study with a Chinese cohort, although the CST 2 and CST5 categories were less common, making up in 2.18% and 1.75% of the vaginal samples, respectively (15).

*L. crispatus* strains in the vaginal microbiota, referred to as CST 1, seem to have protective effects through bacteriocin, toxin‒antitoxin systems, and other functional elements in vaginal secretions, increasing the susceptibility of the vaginal barrier to genital infections (10). The dominance of *L. crispatus* in the vaginal microbiota is associated with a lower prevalence of HPV, human immunodeficiency virus (HIV) and herpes simplex virus type 2 (HSV-2) infection (16). We found a slightly lower number of HPV-positive samples in the CST 1 group. In terms of cytological classification, in the CST 1 category, negative cytology was found in 26.3% of samples, followed by ASCUS in 21.1% of samples, LSIL in 36.8% of samples, and HSIL in 15.8% of samples. Among the eleven biopsies from the CST 1 group, eight were negative for the presence of cervical precancerous conditions. Despite the small number of biopsies performed, these findings also suggest a protective role of *L. crispatus* in cervical carcinogenesis.

Indeed, *L. gasseri*, referring to the CST 2 category, can play a role in maintaining vaginal health and might be helpful in HPV clearance (17). Its possible protective role in the vaginal microbiota was also noted in our study. We observed the dominance of NILM cytology in the CST 2 category, while negative cytology was present in 45.4% of cervicovaginal samples from patients with *L. gasseri* dominance. The remaining cytological categories were found in descending order of lesion severity: HSIL in 27.3% of samples, LSIL in 18.2% of samples, and ASCUS in 9.1% of samples. Most CST2 samples were HPV negative (63.6%). Analysis of the coexistence of *L. iners* with other bacteria across all CST types revealed a three-fold (OR = 0.333) lower significance (*p* = 0.022, adj. *p* val = 0.131) of coexistence of *L. gasseri* with *L. iners*, which may highlight the protective role of *L. gasseri*. However, the cohort of patients in the CST 2 category was not large enough to draw specific conclusions.

The CST 3 group is known to be dominated by *L. iners*. Owing to probable rapid evolution events, with large-scale gene loss, *L. iners* has the smallest genome on a single chromosome among *Lactobacillus* spp. It is speculated that the small size of the genome may be indicative of a symbiotic or parasitic lifestyle in comparison with other lactobacilli and that this species cannot adequately adapt to changes in environmental conditions (10, 18). Some researchers have revealed a tendency of the *L. crispatus-*dominated vaginal microbiota to change to *L. iners* dominance. Researchers also analysed the similarity between the *L. crispatus* and *L. iners* genomes. Some genes in the *L. iners* genome, such as *Chlamydia*, *Streptococcus*, *Parvimonas*, *Gardnerella* and *Atopobium*, are likely acquired horizontally from bacterial species. Researchers have also speculated that *L. crispatus* may be able to grow under sufficient nutrient conditions, that the vaginal microbiota switches from *L. crispatus-*dominated to *L. iners-*dominated when nutrients become insufficient and that cytolysin is essential for liberating *L. iners* from host tissues (18). We detected the coexistence of *L. iners* with *L. crispatus* in 46.2% (12/26) of samples in the CST3 group and in 47.4% (9/19) of samples in the CST1 group. The CST3 group typically presented greater bacterial diversity (Shannon index, 0.47) and more HPV-positive samples (73.1%), suggesting that the cervical epithelium of *L. iners* was less protected. This finding corresponds with the known abundance of *L. iners* in HPV-infected women, and its presence may contribute to the maintenance of vaginal dysbiosis (19).

Owing to the absence of genes, *L. iners* is unable to produce the D-lactic acid isoform, which could be one of the possible explanations for its relationship with increased BV prevalence (10). *L. iners* may increase the adhesion of *G. vaginalis*, thereby facilitating BV (20). In our work, *G. vaginalis* was present in 69% of the CST3 samples. Other bacteria associated with BV, such as *U. parvum* and *P. bivia*, were confirmed in more than 30% of the CST 3 samples. We noted that *L. iners* more likely coexisted with *G. vaginalis* and *U. parvum* when bacteria were analysed across all CST types. These results suggest that *L. iners* can be associated with BV, which can play a role in cervical carcinogenesis, while a positive association between BV and cervical precancerous lesions is known (21).

Corresponding with the findings of Norenhag J. et al. (22), our work revealed the presence of HPV infection in 73.1% of CST 3 samples, which was significantly different (*p* = 0.038) from those of the CST 1, 2 and 5 categories, indicating the presence of protective vaginal microbiota in these 3 categories. We also observed that the number of patients in cytological categories increased with increasing severity of cervical dysplasia among women with *L. iners* vaginal microbiota dominance. An investigation of biopsies from the CST 3 category revealed an increasing trend in lesion severity, which may highlight the potential role of *L. iners* in cervical carcinogenesis. This finding is also in line with other studies that considered *L. iners* in the vaginal microbiota as a risk factor for cervical dysplasia (23, 24). Similarly, Oh H.Y. et al. (25) demonstrated an increased risk of CIN in women whose vaginal microbiota was dominated by *L. iners* and in which other bacteria, such as *A. vaginae* and *G. vaginalis*, which are associated with BV, were present (25).

Cervical samples with no *Lactobacillus* spp. dominance are categorised into the CST 4 class, also known as BV, which is the most common vaginal disorder among reproductive-aged women. It is associated with the presence of anaerobic bacteria, depletion of lactobacilli and increased bacterial species diversity (26). A systematic review of a Latina population revealed 24 unique bacteria associated with abnormal cervical cytology. *Sneathia* spp., *Chlamydia trachomatis*, and *G. vaginalis* were consistently enriched in women with abnormal cervical cytology (27). The CST 4 class in our study was characterised by the dominance of more bacterial strains in the samples. The most dominant bacterium was *G. vaginalis*, which was less abundant in 26% of the CST 4 samples. Other dominant strains were *L. amnionii* (13%), *S. agalactiae* (10%), *A. vaginae* (7%), *Candida* spp. (7%), *P. bivia* (7%), and *S. mitis* (7%). We did not confirm any associations with HPV infection or worse biopsy outcomes. Relative bacterial abundance differed significantly across CST classes (Kruskal–Wallis test, adjusted *p* = 0.0012), with the highest Shannon index observed in CST 4 (0.98). We also observed a trend of increasing cytological severity of cervical lesions, although the increase was not significant.

*L. amnionii* can be found in normal vaginal flora and in women with BV (28). However, its high prevalence, along with the presence of *A. vaginae* and *S. sanguinegens*, is linked to spontaneous abortion in women (29).

*S. agalactiae*, or Group B Streptococcus (GBS), is a commensal bacterium typical in the vaginal flora. Some authors have shown its ability to promote *G. vaginalis* biofilm formation in coculture scenarios (30). When GBS is present in the vaginal secretions of pregnant women, it may cause severe neonatal infections that can lead to early illness, such as pneumonia, sepsis or meningitis. Maternal GBS carriage is associated with ectocervical inflammation and contact bleeding (31). Furthermore, *Candida* spp. is also frequently present in the mucosal flora of healthy women and can develop into vulvovaginal candidiasis (VVc) under suitable conditions. VVc can be reduced by recolonisation of the vaginal microflora by probiotic cultures, such as *Lactobacillus* spp (32). Like *G. vaginalis*, *P. bivia* also produces multiple bacterial sialidase enzymes that were also isolated from the genera *Bacteroidales*, *Bacteroides*, *Bifidobacterium*, *Corynebacterium* (Actinomycetia), and *Streptococcus* and are linked to negative health outcomes, such as BV and preterm birth (33).

One of the most typical bacteria of the BV environment is *G. vaginalis* (34). According to the VALENCIA classifier, CST 4 can be divided into CST 4-A, with a high relative abundance of *Candidatus Lachnocurva vaginae* and a moderate relative abundance of *G. vaginalis*, and CST 4-B, characterised by a high relative abundance of *G. vaginalis* and a low relative abundance of *Ca. L. vaginae*; CST 4-A and CST 4-B are both characterised by moderate relative abundances of *A. vaginae* (35). Furthermore, VALENCIA splits CST 4-C, characterised by a low relative abundance of *Lactobacillus* spp., *G. vaginalis*, *A. vaginae*, and *Ca. L. vaginae*, into 5 sub-CSTs, whereas CST IV-C1 is dominated by *Streptococcus* (35).

According to the VALENCIA classification, *G. vaginalis* dominated 57% of the CST 4 samples, and these samples should be classified as CST 4-B. The coexistence of *A. vaginae* was approximately 11.5-fold greater (raw *p* value = 0.0058; adjusted *p* value = 0.1043) in the presence of *G. vaginalis* than in the absence of *G. vaginalis*. There was a 25-fold (OR = 0.041) lower chance of the presence of *S. agalactiae* (raw *p* value = 0.0312; adjusted *p* value = 0.2812) when *G. vaginalis* was present in the sample than when *G. vaginalis* was absent. This finding is inversely correlated with the results of the VALENCIA CST IV-C1 subgroup, which was characterised by low *S. agalactiae* abundance in the presence of *G. vaginalis*.

Our study suggests that *L. crispatus* and *L. gasseri* play protective roles in maintaining vaginal health. On the other hand, we noted the presence of HPV infection in 73.1% of the CST 3 samples, and the abundance of severe cytologically and histologically confirmed cervical lesions increased with the increasing abundance of vaginal microbiota samples dominated by *L. iners*. Therefore, we suggest that despite the classification of *L. iners* into the genus *Lactobacillus*, which is generally considered protective, its presence may have a negative impact on the development of cervical dysplasia. Our work also revealed statistically significant *G. vaginalis* and *U. urealyticum* coexistence with *L. iners*, whereas other bacterial species were also present in the CST 3 category. We found significantly greater bacterial diversity in the CST 4 class. An increasing trend of bacterial diversity with increasing cytological severity of cervical lesions was also observed, although the increase was not significant. This study also determined the microbial composition of cervical swabs across CST categories and tested its associations with HPV. These findings may indicate that the vaginal microbiota is a modifiable factor of cervical carcinogenesis.

This study has also several limitations. The overall sample size was modest and unevenly distributed across CST groups, with a particularly small number of CST V samples that was excluded from subsequent analysis or was merged with biologically comparable groups. However, appropriate statistical methods suitable for small group sizes were applied to minimize the risk of biased estimates. In addition, while targeted qPCR does not capture the full microbial diversity or provide relative abundance in a true metagenomic sense, it enabled sensitive and quantitative assessment of predefined, clinically relevant bacterial species. On the other side, the selected taxa were based on prior broader screening, and the use of pre-designed qPCR panels enhances potential applicability in routine laboratory diagnostics.

Our article highlights the possible role of *L. iners* in the development of cervical dysplasia. We also found the association of *L. iners* with anaerobic bacteria and indicate the potential role of bacterial diversity in cervical carcinogenesis. We suggest that a correct vaginal microbiota composition can be a protective factor against the development of cervical dysplasia. Current knowledge also highlights the role of the vaginal microbiota in other aspects of health, such as the association of vaginal dysmicrobia with adverse perinatal outcomes and its impact on postnatal development.

## Data Availability

The original contributions presented in the study are included in the article/Supplementary Material, further inquiries can be directed to the corresponding author.
