# Supplementary material for: Lactobacillus iners and vaginal microbiota diversity as risk factors of uterine cervix dysplasia: a prospective study
Source: Front Reprod Health. 2026 Mar 27;8:1797643. doi: 10.3389/frph.2026.1797643 (PMC13066258; doi:10.3389/frph.2026.1797643)
Supplement: Supplementary file 1 [file Datasheet1.docx]

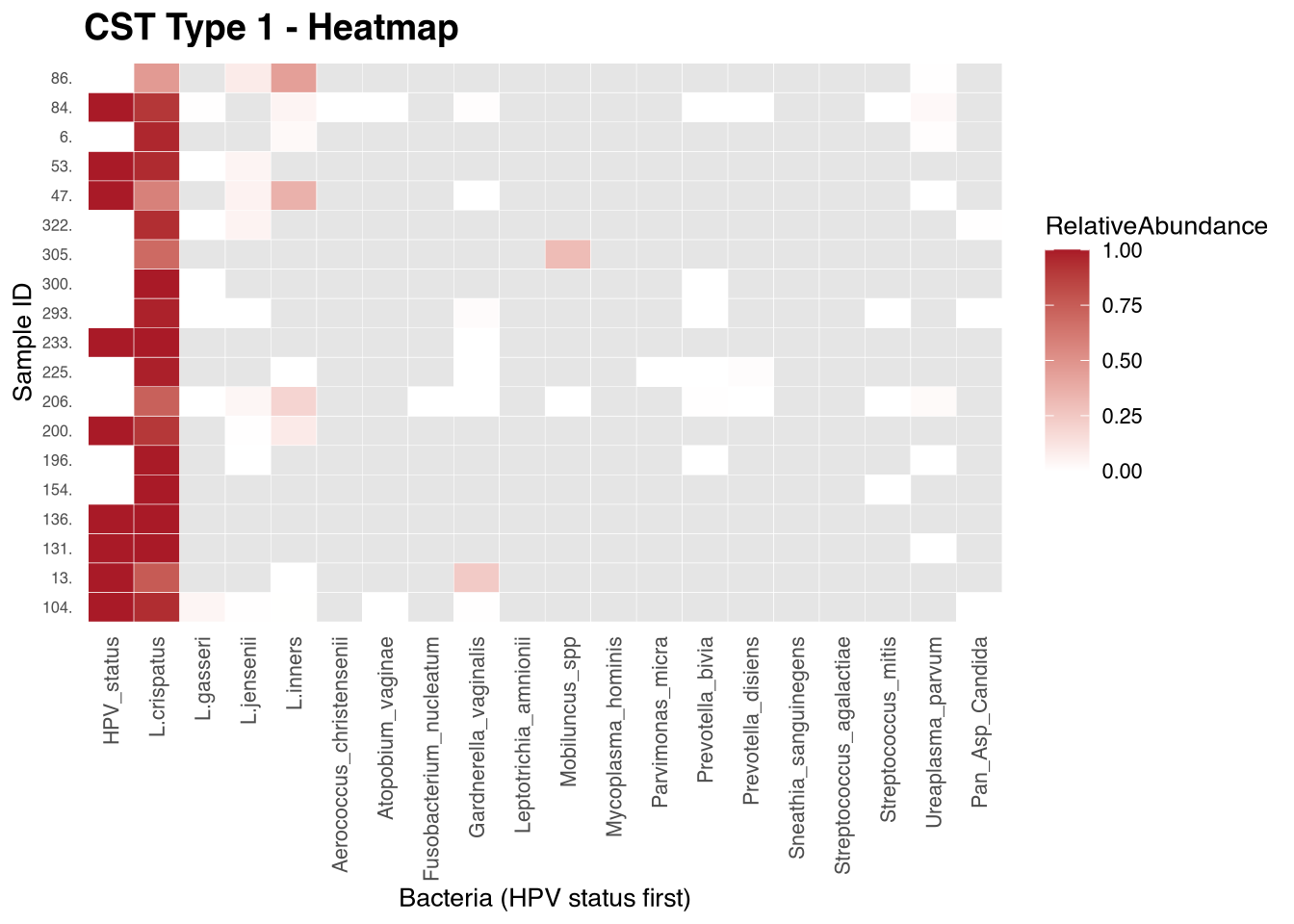


Fig. 2 Heatmap showing the relative abundance of selected bacterial strains and the presence of HPV infection in the CST 1 category. The more intense the red colour means the higher the abundance


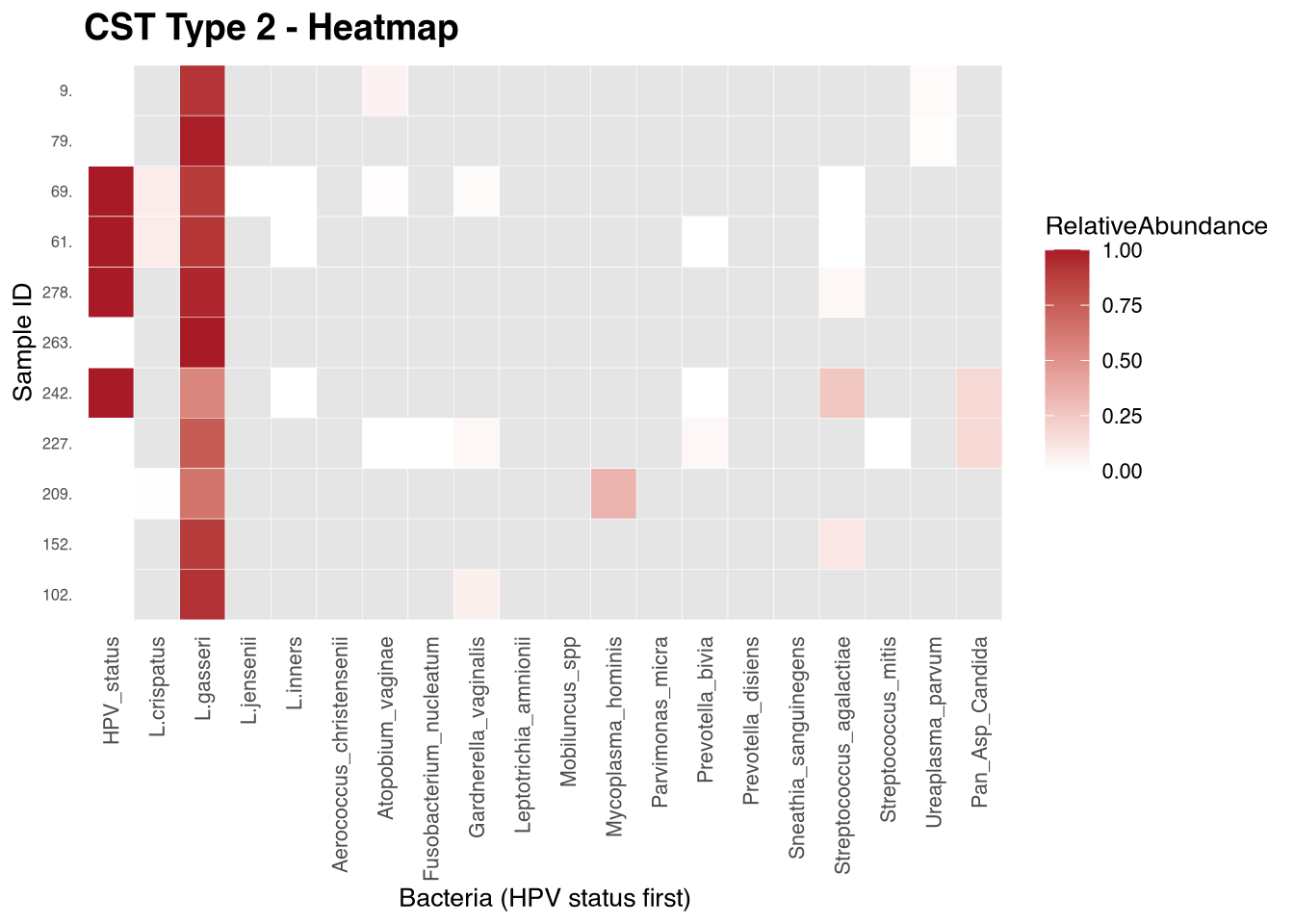


Fig. 3 Heatmap showing the relative abundance of selected bacterial strains and the presence of HPV infection in the CST 2 category. The more intense the red colour means the higher the abundance.


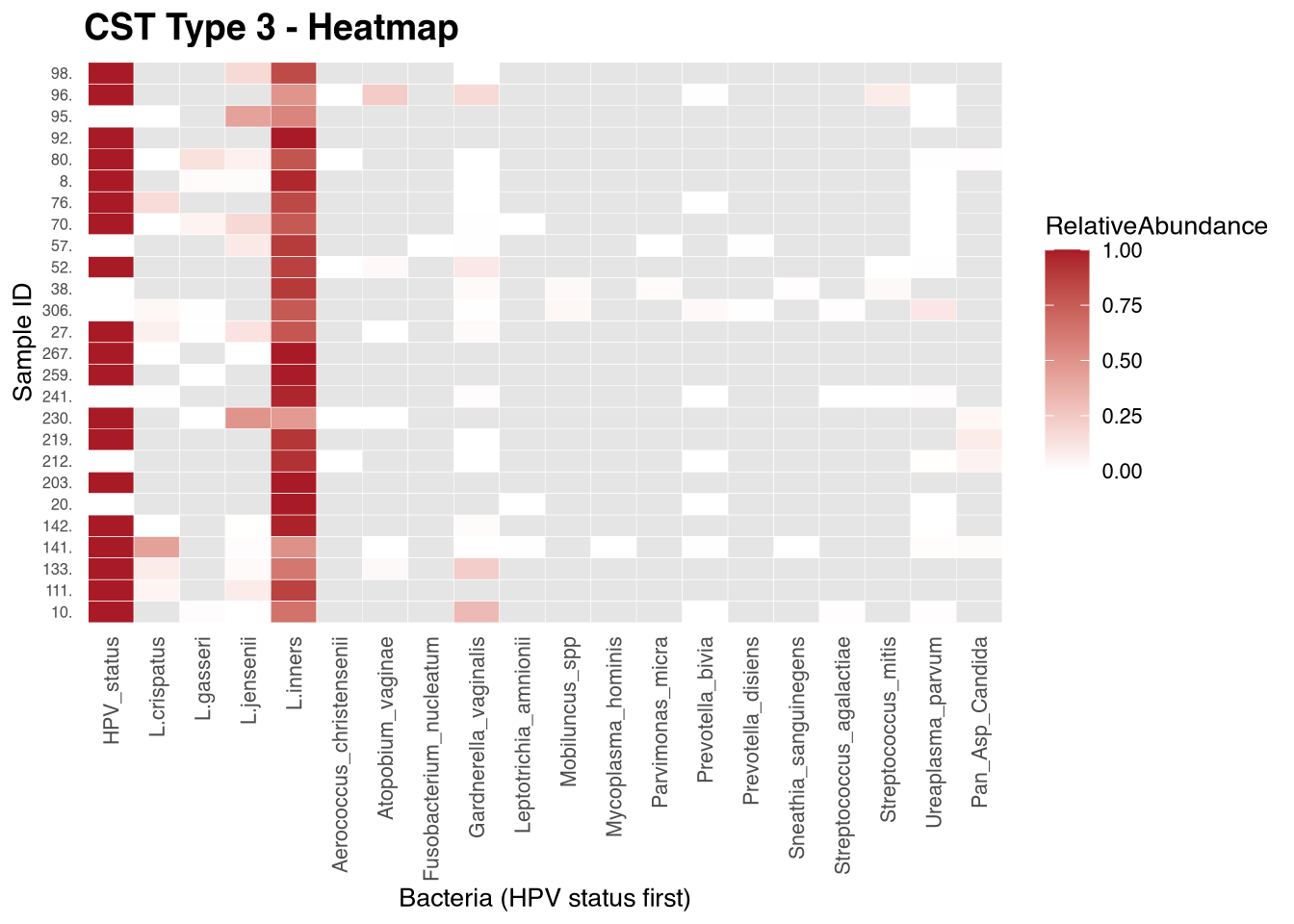


Fig. 4 Heatmap showing the relative abundance of selected bacterial strains and the presence of HPV infection in the CST 3 category. The more intense the red colour means the higher the abundance.


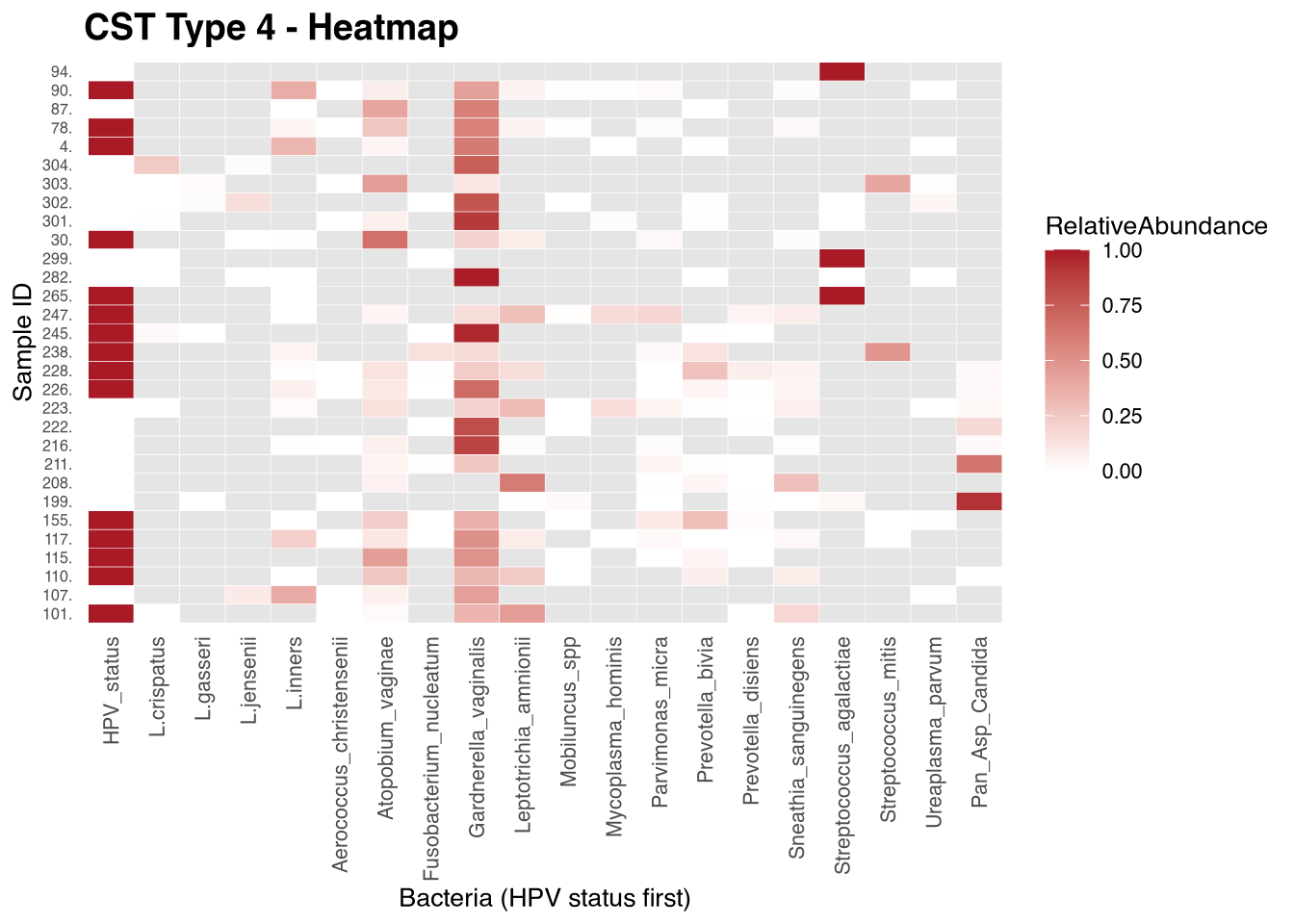
Fig. 5 Heatmap showing the relative abundance of selected bacterial strains and the presence of HPV infection in the CST 4 category. The more intense the red colour means the higher the abundance


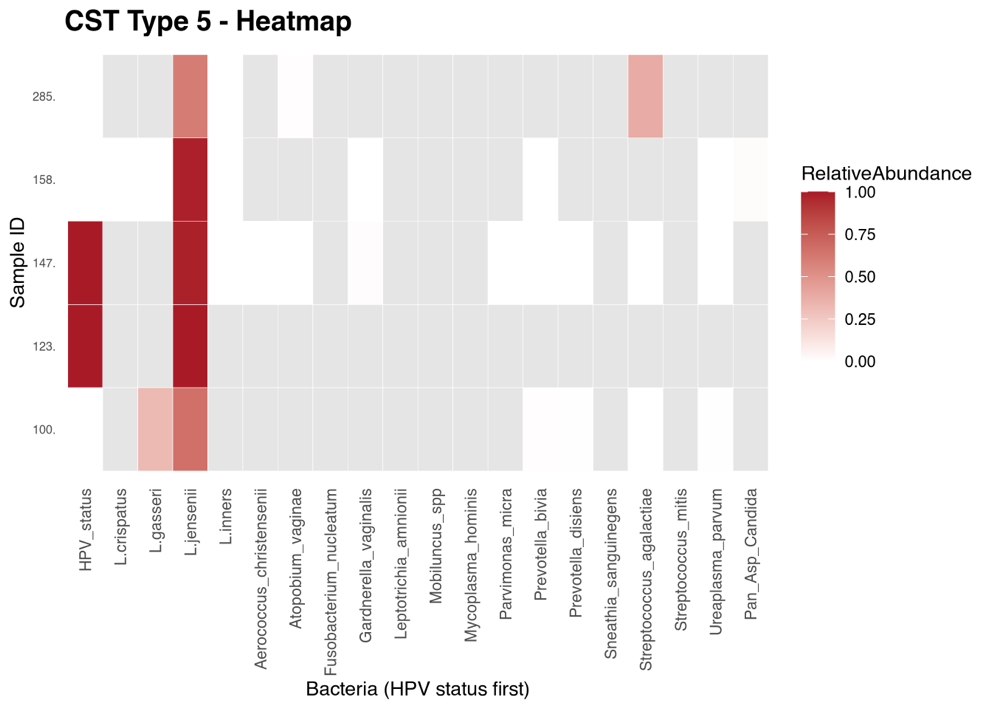


Fig. 6 Heatmap showing the relative abundance of selected bacterial strains and the presence of HPV infection in the CST 5 category. The more intense the red colour means the higher the abundance
